# Supplementary material for: HIVprotI: an integrated web based platform for prediction and design of HIV proteins inhibitors
Source: J Cheminform. 2018 Mar 9;10:12. doi: 10.1186/s13321-018-0266-y (PMC5845081; doi:10.1186/s13321-018-0266-y)
Supplement: Supplementary file 2 — Additional file 2. Source code of HIVProtI web server. [file 13321_2018_266_MOESM2_ESM.zip › HIVprotI_Source-code/hivprotI/weka-3-6-6/doc/weka/core/MultiInstanceCapabilitiesHandler.html]

MultiInstanceCapabilitiesHandler


---


|  |  |  |
| --- | --- | --- |
| **Overview** \| **Package** \| **Class** \| **Tree** \| **Deprecated** \| **Index** \| **Help** \| **Weka's home** | |  |
| **PREV CLASS**   **NEXT CLASS** | **FRAMES**    **NO FRAMES**     **All Classes** |  |
| SUMMARY: NESTED \| FIELD \| CONSTR \| METHOD | DETAIL: FIELD \| CONSTR \| METHOD |  |


---


## weka.core Interface MultiInstanceCapabilitiesHandler

**All Superinterfaces:**: CapabilitiesHandler

**All Known Implementing Classes:**: CitationKNN, MDD, MIBoost, MIDD, MIEMDD, MILR, MINND, MIOptimalBall, MIPolyKernel, MIRBFKernel, MISMO, MISVM, MIWrapper, MultiInstanceToPropositional, SimpleMI

---

``` public interface MultiInstanceCapabilitiesHandler extends CapabilitiesHandler ```

Multi-Instance classifiers can specify an additional Capabilities object
for the data in the relational attribute, since the format of multi-instance
data is fixed to "bag/NOMINAL,data/RELATIONAL,class".

**Version:**
:   $Revision: 1.1 $

**Author:**
:   fracpete (fracpete at waikato dot ac dot nz)

---

| **Method Summary** | |
| --- | --- |
| `Capabilities` | `getMultiInstanceCapabilities()`             Returns the capabilities of this multi-instance classifier for the relational data (i.e., the bags). |

**Methods inherited from interface weka.core.CapabilitiesHandler**

`getCapabilities`

**Method Detail**

### getMultiInstanceCapabilities

```
Capabilities getMultiInstanceCapabilities()
```

:   Returns the capabilities of this multi-instance classifier for the
    relational data (i.e., the bags).

    :   **Returns:**: the capabilities of this object **See Also:**: `Capabilities`


---


|  |  |  |
| --- | --- | --- |
| **Overview** \| **Package** \| **Class** \| **Tree** \| **Deprecated** \| **Index** \| **Help** \| **Weka's home** | |  |
| **PREV CLASS**   **NEXT CLASS** | **FRAMES**    **NO FRAMES**     **All Classes** |  |
| SUMMARY: NESTED \| FIELD \| CONSTR \| METHOD | DETAIL: FIELD \| CONSTR \| METHOD |  |


---
